# Soluble CD14 and IL-12p70 on early antiretroviral therapy predict HIV load setpoint after treatment interruption

**DOI:** 10.1097/QAD.0000000000004159

**Published:** 2025-02-13

**Authors:** Pien M. van Paassen, Anders C. Boyd, Alexander O. Pasternak, Irma Maurer, Agnes M. Harskamp, Marlous L. Grijsen, Suzanne Jurriaans, Jan M. Prins, Neeltje A. Kootstra, Godelieve J. de Bree

**Affiliations:** a Laboratory for Viral Immune Pathogenesis, Department of Experimental Immunology, Amsterdam UMC, University of Amsterdam; b Amsterdam Institute for Immunology and Infectious Diseases, Amsterdam; c HIV Monitoring Foundation; d Laboratory of Experimental Virology, Department of Medical Microbiology, Amsterdam UMC, University of Amsterdam, Amsterdam, The Netherlands; e Oxford University Clinical Research Unit Indonesia (OUCRU ID), Faculty of Medicine Universitas Indonesia, Jakarta, Indonesia; f Centre for Tropical Medicine and Global Health, Nuffield Department of Medicine, University of Oxford, UK; g Department of Internal Medicine, Division of Infectious Diseases, Amsterdam UMC, Amsterdam, The Netherlands.

**Keywords:** antiretroviral therapy interruption, biomarkers, inflammation, set point, viral rebound

## Abstract

**Objective:**

After analytical treatment interruption (ATI), viral rebound occurs in most people with HIV. The time to viral rebound (TTVR) is likely determined by the properties of the viral reservoir as well as the antiviral immune response. Soluble biomarkers of immune activation may be predictive of TTVR and plasma viral load (pVL) setpoint after ATI. The objective of this study is to identify these soluble biomarkers.

**Design:**

A retrospective biomarker analysis of the Primo-SHM trial who were treated 24 or 60 weeks during early HIV infection.

**Methods:**

Thirty-five biomarkers were measured at ATI in 65 participants. Association between biomarkers and reservoir size, TTVR and pVL at setpoint was assessed.

**Results:**

SCD14 correlated to pVL setpoint (*B* = 0.598; *P* = 0.004) and lower levels of IL-12p70 to a higher level of pVL setpoint (*B* = −0.448; *P* = 0.04). SCD163 correlated to levels of total HIV-DNA (*B* = 0.413; *P* = 0.007).

**Conclusion:**

An increased pVL setpoint was associated with higher sCD14 and lower IL-12p70 at ATI, and increased total HIV-DNA was associated with higher sCD163 at ATI. SCD14 and sCD163 are biomarkers of monocyte activation, whereas IL-12p70, produced by monocytes, is essential for inducing Th1 responses. This underscores the relation between immune activation and diminished immune control of HIV. Our findings indicate that sCD14 and IL-12p70 could serve as predictive biomarkers for favorable outcomes in cure interventions.

## Introduction

Persistence of the HIV reservoir is the major barrier to cure interventions. The clinical endpoints in HIV cure trials that aim to reduce the reservoir are time to viral rebound (TTVR) and plasma viral load (pVL) setpoint after analytical antiretroviral therapy (ART) interruption (ATI). So far, these trials have demonstrated varying degrees of success, with different durations of post-ATI viral control. Despite this, most trial participants still experience viral rebound. To mitigate the risk of viral rebound, it is essential to develop biomarker profiles that identify participants with immunological or virological characteristics associated with a higher likelihood of prolonged viral control after ATI [1]. Recent studies suggest that the TTVR and pVL setpoint after ATI are determined by the constellation of the viral reservoir, immune response, and host genetic factors. In this context, soluble biomarkers that reflect activation of the immune system are of specific interest, as they can be readily measured [2].

## Materials and methods

### Study design

In the present study, we explored biomarkers related to immune activation in the Primo-SHM trial, a multicenter, open-label randomized controlled trial that was initiated in 2003 at the Academic Medical Center in Amsterdam in which individuals diagnosed with early HIV infection were randomized to 24 weeks of ART (arm 1), 60 weeks of ART (arm 2) or deferred ART (not included in the present study). Trial methodology and outcomes, as well as treatment regimen and follow-up visits, have been described previously [3]. The time point selected for the present study is ATI (defined as the sampling time point closest to interruption of ART). We investigated the association between immune activation, through 35 cytokines measured immediately before ATI, measures of the viral reservoir and both TTVR (days between ATI and pVL of >200 copies/ml) and pVL setpoint (defined as the pVL at 36 weeks after ATI). Blood samples were obtained in accordance with the protocol approved by the Institutional Review Board of the Academic Medical Center and all participants provided written, informed consent.

### HIV viral load

Plasma viral loads were measured using a sensitive HIV-RNA assay with a lower limit of detection of 40 or 50 copies/ml [4]. The assays that were used, depending on the study site, were Amplicor HIV Monitor ultrasensitive RNA assay (Roche, Basel, Switzerland), Amplicor HIV Monitor, Cobas Amplicor, Cobas TaqMan HIV (Roche Diagnostics), m2000rt HIV-RNA (Abbott, Abbott Park, Illinois, USA), NucliSens EasyQ (bioMérieux, Marcy-l’Étoile, France), Quantiplex bDNA 3.0 (Chiron, Corporation, Emeryville, California, USA), and Versant HIV-RNA 3.0 Assay (Siemens Healthcare Diagnostics, Deerfield, Illinois, USA) [3].

### Viral reservoir measurements

Cell-associated (CA) HIV-RNA and CA HIV-DNA assays have been described previously [5]. In brief, total nucleic acids were extracted from peripheral blood mononuclear cells (PBMCs) using the Boom isolation method [6]. CA HIV-RNA and CA HIV-DNA were measured using previously described quantitative PCR-based (qPCR-based) methods [7,8]. HIV-DNA or RNA copy numbers were determined using a standard curve and normalized to the total cellular DNA (by measurement of β-actin DNA) or RNA (by measurement of 18S ribosomal RNA) inputs, respectively, as described previously [9].

### Plasma biomarker analysis

ELISAs were used to determine the plasma levels of I-FABP, sCD14, sCD163 (R&D systems, Minneapolis, Minnesota, USA; duoset ELISA), retinoic acid (RA; Cusabio Technology, Houston, Texas, USA), hyaluronic acid (HA; Cloud-Clone Corp, Houston, Texas, USA), sTNF-RI (BioSource Europe S.A., Nivelles, Belgium), TGF-β, CRP, Rantes, (e-Bioscience, San Diego, California, USA). Luminex was used to determine the plasma levels of D-Dimer, IFN-γ, IFN-α, IFN-β, IL-1β, IL-1Rα, IL-10, IL-12p70, IL-13, IL-17α, IL-2, IL-21, IL-27, IL-4, IL-5, IL-6, IL-7, IL-8, IP-10, MCP-1, MIG, TNF-α, sCD40L GM-CSF, IL-12p40, and MIP-1α (Thermo Fisher Scientific, Waltham, Massachusetts, USA; PPX-23-MXTZ76X and PPX-03-MXPRJZ3). ELISAs and Luminex were performed according to the manufacturers’ instructions.

### Statistical analysis

Inflammatory markers were log_10_-transformed and summarized as box-plots and stratified by treatment arm. To assess the relationship between biomarkers, correlations were determined using Pearson's tests and visualized in networks using the ‘corrplot’ package in R studio [10]. The mean pVL set point and mean viral reservoir size (i.e. total HIV-DNA and CA HIV-RNA) were modeled using linear regression. Cox regression analysis was performed for the relationship between biomarkers and TTVR after ATI. Statistical analyses were performed with STATA (v15.1, College Station, Texas, USA) and R (v4.2.1, Vienna, Austria). Significance was defined as a *P* value less than 0.05.

## Results

**Fig. 1:**
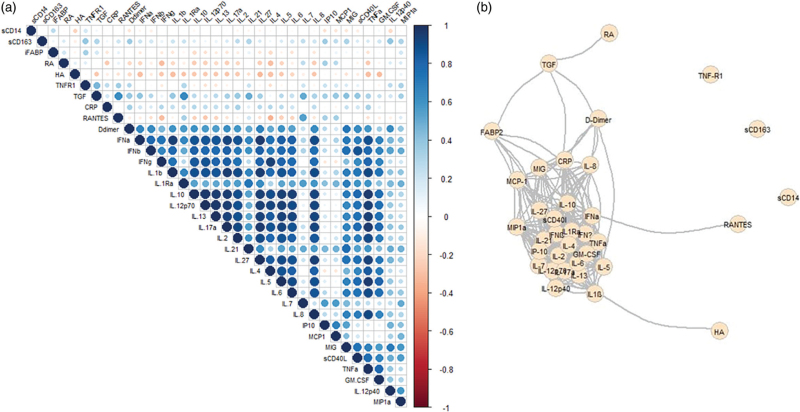
Correlation between plasma biomarkers.

**Fig. 2:**
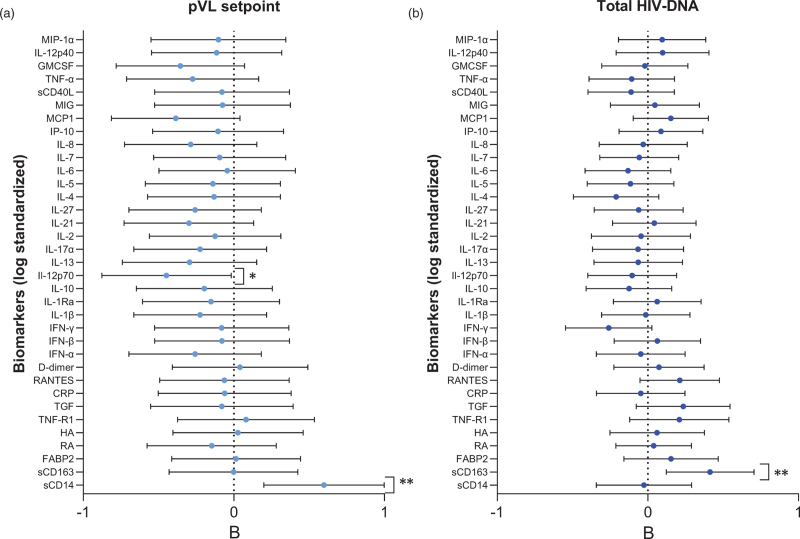
Associations of biomarkers with viral load set point (a) and total HIV-DNA (b).

Previously, it was found that after ATI, participants in arms 1 and 2 experienced a significantly lower pVL setpoint and higher CD4^+^ T-cell count compared to the deferred arm [3]. In the present study, 65 participants (arm 1: *n* = 29, arm 2: *n* = 36) were included. Most participants (60/65) had an undetectable pVL at ATI. Of the five participants with detectable pVL at ATI, two had a pVL below 100 copies/ml and three had a pVL between 977–1882 copies/ml. First, the levels of individual biomarkers were compared between arms 1 and 2, and no differences were observed in biomarker levels between the two treatment arms. Therefore, both arms were combined in subsequent analyses. Given the potential interdependence between biomarkers, we used Pearson's correlation to investigate the correlations between biomarker levels (Fig. 1a). Indeed, correlations between biomarkers of coagulation, inflammation, and immune activation were observed. The biomarkers sCD14, sCD163, and TNF-R1 did not, however, correlate with any of the other biomarkers (Fig. 1b). Next, we analyzed the association between the biomarkers individually, TTVR and pVL setpoint using Cox regression and linear regression. TTVR was only assessed for those with an undetectable pVL at ATI. SCD14 levels in the upper 75%tile, indicative of higher inflammation, were associated with a higher mean pVL setpoint [*B* = 0.6, 95% confidence interval (CI) 0.2–1.0; *P* = 0.004], whereas IL-12p70 levels in the upper 75%tile were associated with a lower mean pVL at setpoint (*B* = −0.45; 95% CI −0.88 to 0.02; *P* = 0.04) (Fig. 2a). No significant associations between individual biomarkers and TTVR were found.

(a) Depicted are correlations between plasma biomarkers. In blue are the positive correlations and in red the negative correlations as determined by Pearson's correlation test. (b) Correlation network based on Pearson's correlation. The closer nodes represent stronger correlations (threshold 0.10). Distinct markers in this network of biomarkers are sCD14, sCD163 and TNF-r1. No gray lines connect these three markers as correlations with all other makers were never above 0.10.

Depicted by dots, is the mean change in outcome per one-unit increase in log_10_-standardized biomarker. Lines depict 95% confidence intervals. (a) The level of IL-12p70 was negatively (*B* = −0.45; 95% CI −0.88 to 0.02; *P* = 0.04) associated with the height of viral load at set point, whereas the level of sCD14 positively (*B* = 0.6, 95% CI 0.2–1.0; *P* = 0.004) associated with height of viral load at set point (right). (b) Levels of sCD163 were positively correlated with total HIV-DNA (*B* = 0.41, 95% CI 0.12–0.71, *P* = 0.007). ^∗^*P* < 0.05, ^∗∗^*P* < 0.01.

Measurements of the total reservoir size and transcriptionally active viral reservoir (total HIV-DNA and CA HIV-RNA, respectively) at ATI were available from 44 of these 65 participants (arm 1; *n* = 20, arm 2; *n* = 24). All 44 participants had an undetectable pVL at ATI. The median levels of CA HIV-RNA and total HIV-DNA at ATI were 1.98 [interquartile range (IQR) 1.24–2.7] log_10_ copies/10^6^ PBMC and 2.14 (IQR 1.19–2.6) log_10_ copies/μg total RNA, respectively, and did not differ between the treatment arms [5]. We assessed whether any of the biomarkers was associated with the total or the transcriptionally active HIV reservoir at ATI using linear regression. We observed that participants with sCD163 levels in the upper 75%tile at ATI had higher mean total HIV-DNA (*B* = 0.41, 95% CI 0.12–0.71, *P* = 0.007) (Fig. 2b). Moreover, levels of CA HIV-RNA tended to increase as sCD14 (*B* = 0.26, 95% CI −0.05 to 0.56, *P* = 0.1) and sCD163 (*B* = 0.28, 95% CI −0.03 to 0.6, *P* = 0.07) increased.

## Discussion

SCD14 and sCD163 are biomarkers of monocyte activation and likely regulated by a multitude of factors. In particular, they are driven by residual viral activity and microbial translocation and are a reflection of immune system dysregulation in treated acute HIV infection [11]. The observed positive correlation between sCD14 and pVL at setpoint, as a proxy for immune control of HIV replication, suggests that immune activation at ATI may be predictive for less efficient immune control. The association between sCD163 and total HIV-DNA at ATI suggests a direct relationship between monocyte and macrophage activation and viral reservoir. Indeed, a role of these cells in reservoir formation and persistence has been shown recently [12]. Finally, IL-12p70 is an immune regulatory cytokine produced by monocytes and dendritic cells that is essential in instructing the adaptive Th1 antiviral immune response. The observed inverse correlation between lower levels of IL-12p70 at ATI and a higher level of pVL at setpoint may reflect immune dysfunction leading to a less potent antiviral T-cell response after ATI. Here, we analyzed people who initiated ART during primary HIV infection and were on ART for 24 and 60 weeks. The outcomes of our study could be different for people who are on long-term ART [13,14] or started treatment during chronic HIV infection because of the composition of the viral reservoir and prolonged inflammation and immune dysfunction [15].

Taken together, the results of our study suggest that sCD14 and Il-12p70 at ATI may be of use in predicting favorable outcome (i.e. lower viral set point) upon ATI after an experimental cure intervention.

## Acknowledgements

J.P., G.J.d.B., and N.K. conceived and designed the study. P.v.P., A.P., I.M., A.H., S.J., and M.G. collected the data. N.K., P.v.P., A.B. and G.J.d.B. performed the analysis. N.K., P.v.P., A.B., A.P., J.P., and G.J.d.B. contributed to interpretation of the results. P.v.P., N.K., G.J.d.B., and J.P. wrote the article.

We would like to thank the participants of the Primo-SHM study for their participation.

The Primo-SHM study has been made possible through the collaborative efforts of the Primo-SHM study group.

### Conflicts of interest

A.B. reports receiving speaker's fees from Gilead Sciences, Inc. For the remaining authors, none were declared. The Primo-SHM inflammation study was financially supported by the Dutch Aidsfonds (P-60803).
